# Expression of Transposable Elements throughout the *Fasciola hepatica* Trematode Life Cycle

**DOI:** 10.3390/ncrna10040039

**Published:** 2024-07-03

**Authors:** Elizaveta K. Skalon, Nick V. Panyushev, Olga I. Podgornaya, Anastasia R. Smolyaninova, Anna I. Solovyeva

**Affiliations:** 1 Zoological Institute, Russian Academy of Sciences, 199034 St. Petersburg, Russia; elizavetaskalon@gmail.com; 2 Bioinformatics Institute, 197342 St. Petersburg, Russia; panyushev@nextmail.ru; 3 Institute of Cytology, Russian Academy of Sciences, 194064 St. Petersburg, Russia; opodg@yahoo.com (O.I.P.); sar28sir14@gmail.com (A.R.S.)

**Keywords:** transposable elements, long non-coding RNA, *Fasciola hepatica* life cycle, RNA-seq analysis

## Abstract

Background: Transposable elements (TEs) are major components of eukaryotic genomes. The extensive body of evidence suggests that although they were once considered “genomic parasites”, transposons and their transcripts perform specific functions, such as regulation of early embryo development. Understanding the role of TEs in such parasites as trematodes is becoming critically important. *Fasciola hepatica*, a parasite affecting humans and livestock, undergoes a complex life cycle in diverse environments and hosts, and knowledge about its life cycle regulation is scarce so far. Methods: We summarized the data regarding the repetitive elements in *F. hepatica* and conducted bulk RNA-seq analysis across its life cycle stages. TE expression profiles were analyzed, focusing on differential expression and potential homology with previously described long non-coding RNAs (lncRNAs). Results: Differential expression analysis revealed stage-specific TE transcription patterns, notably peaking during egg and metacercariae stages. Some TEs showed homology with known lncRNAs and contained putative transcription factor binding sites. Interestingly, TE transcription levels were highest in eggs and metacercariae compared to adults, suggesting regulatory roles in trematode life cycle transitions. Conclusions: These findings suggest that TEs may play roles in regulating trematode life cycle transitions. Moreover, TE homology with lncRNAs underscores their significance in gene regulation.

## 1. Introduction

Trematodes, a highly successful group of parasitic flatworms, infect both humans and domestic animals. According to the World Health Organization, millions of people are infected with trematode parasites, with nearly half of the global population at risk of infection [1] (https://www.who.int/news-room/fact-sheets/detail/foodborne-trematode-infections, last accessed 15 April 2024). Trematode infestations can lead to various diseases, including urinary bladder cancer (caused by *Schistosoma mansoni* and *S. haematobium* infections [2,3,4]), cholangiocarcinoma (associated with *Clonorchis sinensis* and *Opisthorchis viverrini* infections [5,6]), and hepatitis (associated with *Fasciola* spp. infections [7]). Better understanding of the trematode life cycle would allow to effectively disrupt trematode development strategy, thus limiting infestations in definitive hosts. 

Trematode life cycles encompass multiple generations occurring in diverse hosts and environments, regulated by intricate mechanisms. However, studies on trematode life cycle regulation remain scarce. Despite the comprehensive characterization of gene expression patterns in individual stages [8,9,10,11,12], the genomes of *Schistosoma mansoni*, *S. japonicum*, and *Fasciola hepatica* have been shown to encode long non-coding RNAs (lncRNAs)—transcripts exceeding 200 nucleotides in length with no discernible protein-coding potential. Recent research has revealed differential expression of lncRNAs in *F. hepatica* and *Schistosoma* spp. [13,14,15], but the exact regulatory roles of these lncRNAs remain undetermined.

LncRNAs can originate from transposable elements or TEs [16,17], which are mobile genetic elements capable of relocating within the genome. TEs come in two main types: retrotransposons and DNA transposons [18,19,20,21]. Retrotransposons propagate throughout the genome using a “copy-paste” mechanism, facilitated by reverse transcription, whereas DNA transposons lack reverse transcription and move via a “cut-and-paste” mechanism. Retroelements, in turn, are divided into retrotransposons that have Long Terminal Repeats (LTRs) and those without LTRs (non-LTR retrotransposons). Non-LTR retrotransposons include LINEs (Long Interspersed Nuclear Elements) and SINEs (Short Interspersed Nuclear Elements), while LTR retrotransposons also include endogenous retroviruses. Due to imperfect transposition mechanisms and accumulated mutations, transposons can degenerate to shortened, mostly inactive sequences [18,19,20,21]. Transposons also vary greatly in size, from thousands of base pairs (retroviruses, Helitrons) to a few hundred base pairs (MITEs, Alu). Although rare [22], TE jumping events can nevertheless impact genome regulation in various ways, such as gene structure disruption, transcription modulation through the TEs’ own regulatory sequences, or by attracting small non-coding RNAs and other regulatory molecules, which facilitates ectopic recombination and affects genome size [23,24,25,26].

*F. hepatica* (Trematoda, Fasciolidae) is a cosmopolitan parasitic flatworm that causes fasciolosis in ruminants and less commonly in humans. The life cycle of *F. hepatica* is realized as follows: adult flukes lay eggs in the bile ducts, which are eventually excreted into the intestines and released into the environment. Here, ciliated embryos known as miracidia hatch from the eggs. The miracidia actively seek out and penetrate mollusks, typically snails of the Lymnaeidae family, serving as intermediate hosts for further development. Once inside the snail, the parasite undergoes asexual reproduction, progressing through rediae and cercariae stages, leading to clonal expansion. Mature cercariae exit the mollusk and rapidly encyst on plant substrates, forming metacercariae, which can remain infective for months. Infection occurs through ingestion of contaminated vegetation, meat, or water. Upon ingestion, the parasites emerge from their cysts in the intestine as newly excysted or newly emerged juveniles (NEJs), perforate the intestine, and migrate to the liver capsule and parenchyma via the peritoneal cavity [27,28].

*F. hepatica* has one of the largest genomes among trematodes. Its genome was sequenced several years ago [27,29], and TEs are estimated to comprise 40% [27] to 50% [29]. Recently, both short and long non-coding RNAs of this fluke have been described [15,30,31], yet the expression of mobile elements remains unexplored.

This research aims to investigate TE expression and identify critical points in the *F. hepatica* life cycle where transcription of mobile elements is likely most significant. In addition, our findings will provide insights into the influence of TEs on transcriptional regulation and the evolution of the trematode genome.

## 2. Results

### 2.1. Repeat Content in the F. hepatica Genome

We analyzed three of the five available *F. hepatica* genome assemblies in the NCBI database (https://www.ncbi.nlm.nih.gov/genome/, last accessed 15 April 2024), including the latest assembly generated from PacBio long reads. During the search, the RepeatModeler2, a tool for repetitive element recognition, assigns a number to each of the repeats found in every round of the search and classifies them, for instance, as rnd-1_family-34#LINE/Penelope. Repeats that RepeatModeler2 was unable to assign to any known family are grouped together, and in the text, they will be designated as Unknown elements.

Each analyzed assembly revealed that repeats constitute 63–66% of the genome, with approximately one-third remaining unidentified (Supplementary File S1, Table S5).

**Figure 1:**
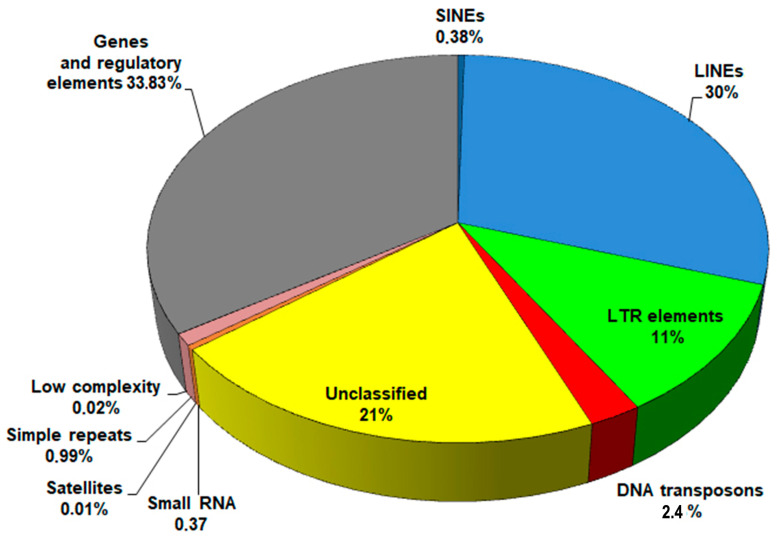
The pie chart showing the mean composition of the *F. hepatica* genome.

Average proportions of genomic sequences are shown in Figure 1. Despite slight variations in repeat content among the genomes, attributed to differences in sequencing technologies and assembly methods, LINEs consistently showed the most abundant presence, accounting for up to 29–30% of the genome.

### 2.2. Transcription of Repetitive Elements and Candidate TEs with Stage-Specific Expression

Repeat masking of the *F. hepatica* transcriptome assembly revealed the utmost abundance of LINEs, comprising 12.04% of assembled transcripts, with Penelope elements included. SINEs occupy 0.76%, DNA transposons account for 0.98%, and unclassified elements make up 6.76%. Thus, 20.54% of the assembled transcripts contain TE sequences. The investigation of solely protein-coding transcripts available at Wormbase (https://ftp.ebi.ac.uk/pub/databases/wormbase/parasite/releases/WBPS19/species/fasciola_hepatica/PRJEB58756/fasciola_hepatica.PRJEB58756.WBPS19.CDS_transcripts.fa.gz, last accessed 15 June 2024) showed that TEs were present in 6.51% of these transcripts, with overwhelming prevalence of LINEs and Unknown elements, presumably due to intronic TEs.

**Figure 2:**
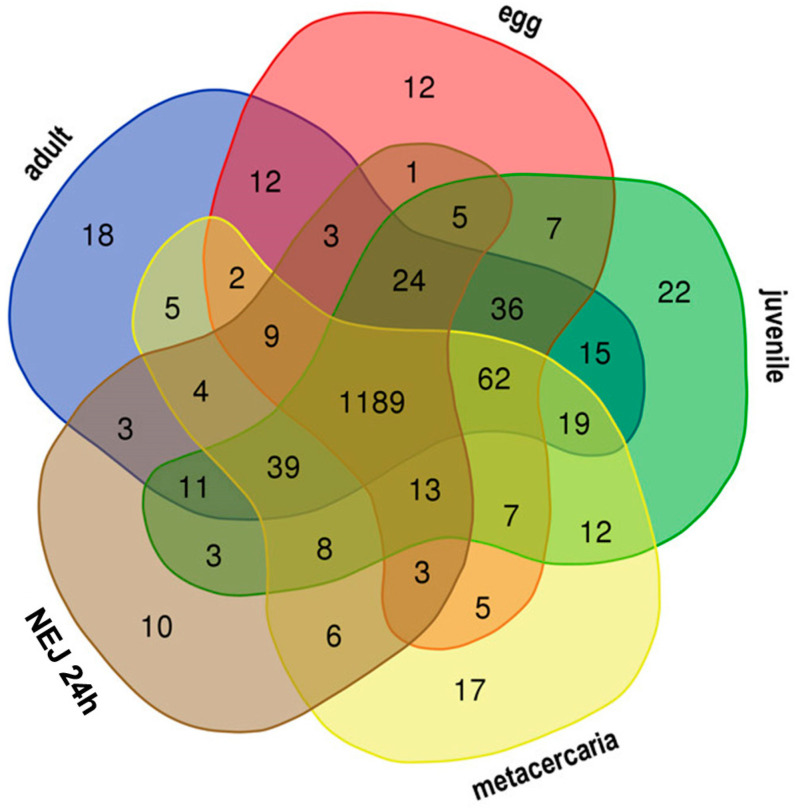
Venn diagram showing candidate TEs with stage-specific expression in *F. hepatica* life cycle. The figures at the intersection of the diagram correspond to the number of commonly transcribed TEs, while the figures outside the intersections show the numbers of stage-specific TEs.

A primary qualitative analysis was performed to investigate the composition of expressed TEs at different stages of the *F. hepatica* life. Kallisto was used to identify TPM (transcripts per million) values for TEs, followed by the comparative analysis of TEs with expression levels over 1 TPM. Figure 2 shows the Venn diagram of transposon expression throughout five stages. Based on the comparative analysis results, Table 1 and Table 2 provide a detailed characterization of stage-specific TE expression profiles. Supplementary Materials (Table S1) include additional information on shared TEs at particular life cycle stages.

The 21-day juvenile stage exhibits the highest number of specific TE transcripts across various comparison combinations, yielding 22 (Figure 2, Table 1 and Table S1) and 19 repeats (Table 2 and Table S1). When compared to the adult stage, metacercariae, eggs, and young juveniles, the majority of specific TEs for the 21-day juvenile stage are represented by Unknown elements, with only five transposons identified as retroelements.

When compared to larvae of other instars (see Table S6 in the Supplementary File S1 and Table S1), the 21-day juvenile is characterized by a greater number of specific TEs and expresses DNA transposons of the CMC-EnSpm and Pogo families. Among the DNA transposons, only the 2355#DNA/TcMar-Tigger is selectively transcribed in the metacercariae, while the remaining stages mainly express Unknown elements and retrotransposons from the CR1, Gypsy, and Pao families. Newly emerged (or excysted) juveniles (NEJs) of 1, 3, and 24 h age exhibit the lowest number of specifically transcribed transposable elements (TEs).

### 2.3. Differential Expression Analysis of Transposable Elements

**Figure 3:**
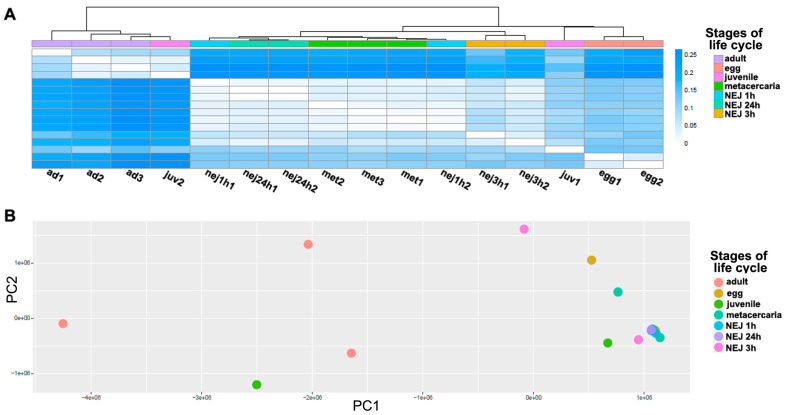
The analysis of TE expression profiles between transcriptomes throughout *F. hepatica* life cycle stages. (**A**) The heatmap shows Jensen–Shannon divergence based on TE expression profiles for each sample; the darker the color, the greater the divergence. (**B**) The principal component analysis (PCA). Based on the TE expression profiles, the dot plot shows the distribution of samples between principal components 1 (*X*-axis) and 2 (*Y*-axis). Ad—adult; egg—egg; juv—21-day juvenile; met—metacercaria; NEJ1 h, 3 h, 24 h—newly emerged juveniles after 1, 3, and 24 h of excystment.

A differential expression analysis was carried out to identify the common features in the TE transcriptional profile of *F. hepatica* stages of the life cycle. The heatmap (Figure 3A) and principal component analysis (PCA, Figure 3B) were used to analyze similarities in the overall TE expression. The PCA revealed clustering of sample replicates and relative variance between samples in principal components 1 and 2 (PC1 and PC2, Figure 3B). Both PCA and Jensen–Shannon distances indicate that there is no clear clustering between stages based on the total transposon transcription. To identify differentially expressed TEs, the following two approaches were implemented: (1) the multiple comparison across all stages using a likelihood ratio test (LRT), and (2) pairwise comparisons of adults with the other stages using the Wald test. As expected, the analysis failed to identify TEs that were exclusively expressed at one stage as differentially expressed (see Table 1 and Table 2).

**Figure 4:**
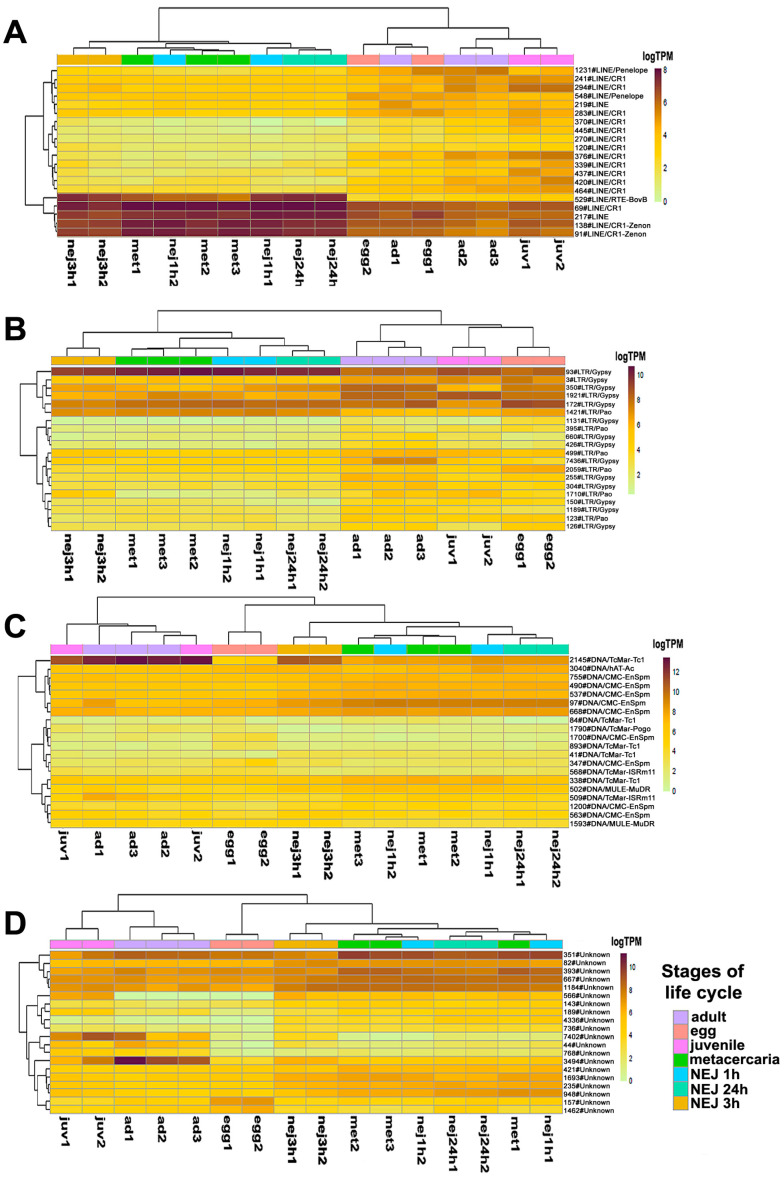
The heatmap of the top 20 differentially expressed transposable elements sorted by *p*-values: (**A**)—LINE, (**B**)—LTR retroelements, (**C**)—DNA transposons, (**D**)—Unknown TE. Colored squares on the top indicate stages of the life cycle. Ad—adult, egg—egg, juv—21-day juvenile, met—metacercaria, NEJ1h, 3h, 24h—newly emerged juveniles after 1, 3, and 24 h after excystment.

The likelihood ratio test (LRT) allowing multiple comparisons across all samples revealed 676 differentially expressed repeats (Table S2, tab sleuth_significant), comprising 320 Unknown elements, 181 LINEs, 2 SINEs, 128 LTR retroelements, 40 DNA transposons, 2 satellites and simple repeats, and 3 tRNA pseudogenes. Figure 4 shows the top 20 differentially expressed transposable elements (TEs), sorted in ascending order of *p*- and q-values of the LRT.

Expression profile analysis of separate TE groups, particularly LTR and Unknown, revealed stage-specific clustering of samples. Several LINEs, including 529#LINE/RTE-BovB, 69#LINE/CR1, 217#LINE, 138#LINE/CR1-Zenon, and 91#LINE/CR1-Zenon, as well as the LTR element 93#LTR/Gypsy, exhibited high expression levels in metacercariae and NEJs (Figure 4). Many transposons showed significant changes in expression levels throughout the metacercaria stage, which coincided with the process of cercariae transformation.

BLAST with a 70% identity threshold was utilized to compare the differentially expressed TEs with the lncRNAs of *F. hepatica* published by McVeigh et al., 2023 [15]. As a result, 2784 lncRNAs matched 564 elements from the *F. hepatica* repeat database, i.e., one transposon is associated with several lncRNAs. Among these TEs, 370 were Unknown, 115 were LINEs, 3 were SINEs,

44 were LTRs, and 32 were DNA-transposons (see Table S2, tab lnc_rna_blast). Our analysis revealed 333 with differential expression among these 564 repeats.

**Figure 5:**
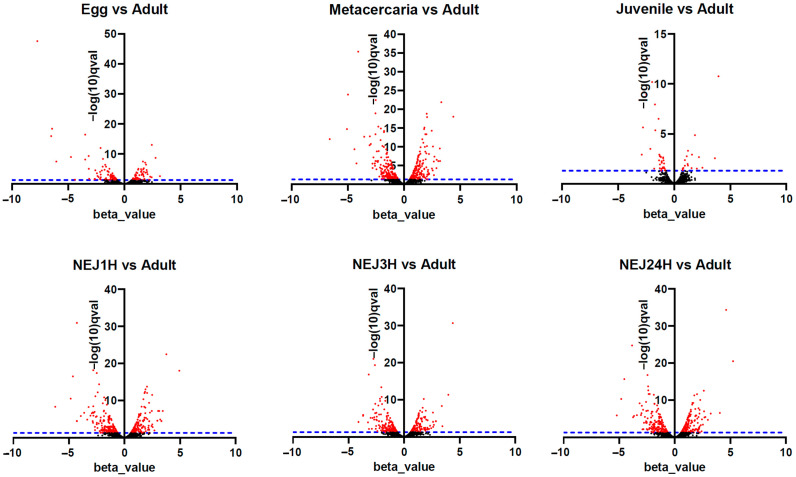
Volcano plots for TE differential expression—adult stage vs. the other stages of the life cycle. The X-axis is a beta value, an analog of fold change, and Y-axis is −log(10)q-val, where qval—q-value, a false discovery rate-adjusted *p*-value in Wald test. The blue dotted line corresponds to q-value < 0.05. Each point in the chart represents a different TE. Black dots indicate false positives, while red dots indicate significant changes in expression. The positive beta value indicates upregulation, and negative is downregulation.

To identify up- or downregulated TEs, we used the Wald test to compare each stage with the marita (adult). Figure 5 represents changes in expression between stages of the life cycle, where each point on the graph indicates a different TE. Such a visualization allows us to observe statistically significant changes in TE expression between life cycle stages.

**Figure 6:**
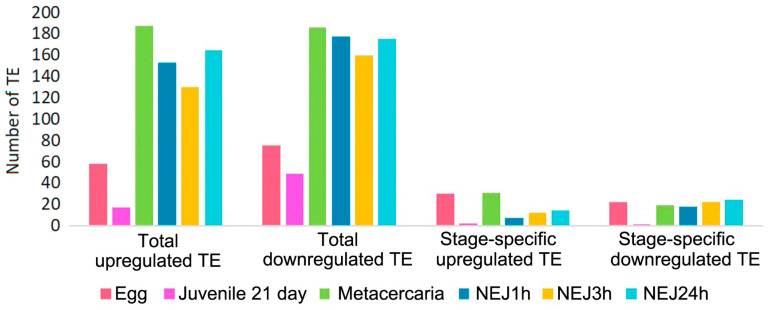
Number of upregulated and downregulated TEs at each stage in comparison to the *F. hepatica* adults. NEJ1h, 3h, 24h—newly emerged juveniles 1, 3 and 24 h after excystment.

Upon initial inspection, metacercariae and NEJs appear to display the greatest number of repeats with significantly differential expression (Figure 5 and Figure 6, Table S3). However, the objective was to identify potential stage-specific repeats with differential expression, i.e., the expression which is highly divergent from the adult and restricted to one particular stage. To this end, sets of differentially expressed TE families (according to the Wald test) were intersected. Venn diagrams were utilized to identify transposons expressed exclusively at one particular stage. The number of TEs is summarized in Figure 6, and the full list of transposons with the stage-specific expression is provided in Table S3 (stage_specific_wald_test tab). Additionally, we tagged TEs with a high identity to lncRNAs, as discovered earlier [15] (see Table S3, stage_specific_wald_test tab, blue cells). As the newly excysted juvenile develops and matures, there are fewer differences in TE expression from the adult stage.

Stage-specific transcription of DNA transposons (Tc1-Mariner, Cmc-EnSpm, Helitron, Pogo) was detected in the egg, metacercariae, and NEJ3h (Table S3). Every analyzed stage was found to express a few specific families of LINE, LTR, and Unknown TE. Owing to the unknown function of TEs in trematodes, defining precise criteria for expression change is challenging; even a small amount of transcript may possess a significant importance for the cell.

However, let us consider a b value (analogous to fold change value) for upregulated stage-specific TEs greater than 1.7, considering that in general, their b values rarely exceed 2 (Table S3). In the egg, ten such elements were identified: 4345#LTR/Gypsy, 420#LINE/Penelope, 1822#LINE/Penelope, 893#DNA/TcMar-Tc1, 956#LINE/L2, and Unknown elements 197#, 610#, 497#, 1252#, and 963#. Metacercariae exhibited five such repeats, respectively (142#DNA/TcMar-Tc1 and Unknown elements 84#, 192#, 408#, 208#), NEJ24h had four Unknown elements (1466#, 3410#, 4521#), while NEJ1h had one (2217#Unknown) and NEJ3h had one (369#DNA/TcMar-Tc1). These TE transcripts could probably be indirectly associated with a possible significant regulatory impact of TEs on the biological processes occurring during specific periods of the parasite life cycle. Thus, the egg and metacercaria stages displayed the highest rates of stage-specific TE expression, along with the highest number of upregulated TEs, corresponding to significant changes to the parasite organism during these stages. In addition, both stages showed the highest number of lncRNA-relative TEs. The egg is characterized by the embryonic development of the miracidium, while the metacercaria undergoes transformation into a young excysted juvenile, which will further migrate from the intestine to the liver, its final localization in the host organism.

It was observed that one of the unclassified TEs, 4336#Unknown (relative to lncRNA), exhibited significantly higher expression levels in metacercariae and NEJ compared to the adult stage (b values > 4). However, in 21-day-old juveniles, the expression level of this transcript did not differ from the adult, though it was downregulated in the egg. Similarly, 529#LINE/RTE-BovB (relative to lncRNA) was upregulated in metacercariae and subsequent stages but not in the egg and 21-day juveniles. Some other transcripts displayed a similar pattern (see Table S3). On the other hand, when compared to the adult stage, some TEs, such as 2145#DNA/TcMar-Tc1 (relative to lncRNA), were significantly downregulated across all investigated stages, except for the 21-day juvenile, and therefore could be potential adult-stage markers.

**Figure 7:**
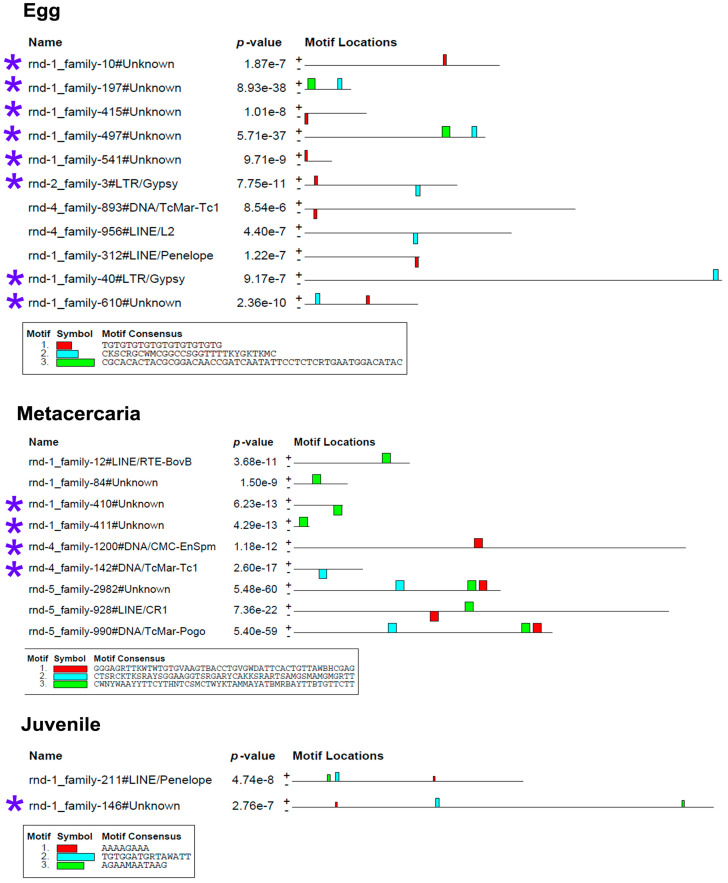
Transcription factor binding site predictions in stage-specific upregulated TE transcripts in egg, metacercaria, and 21-day juvenile. The figure is the layout showing the localization of the identified TF binding sites (colored rectangles) on the forward and reverse TE chain (labelled to the left of the schemes) with the combined match *p*-value. The block height corresponds to the site significance, i.e., taller blocks are more significant. The consensus sequences of the TF binding sites are indicated in boxes under the schemes. Blue asterisks indicate the similar lncRNA TEs.

**Figure 8:**
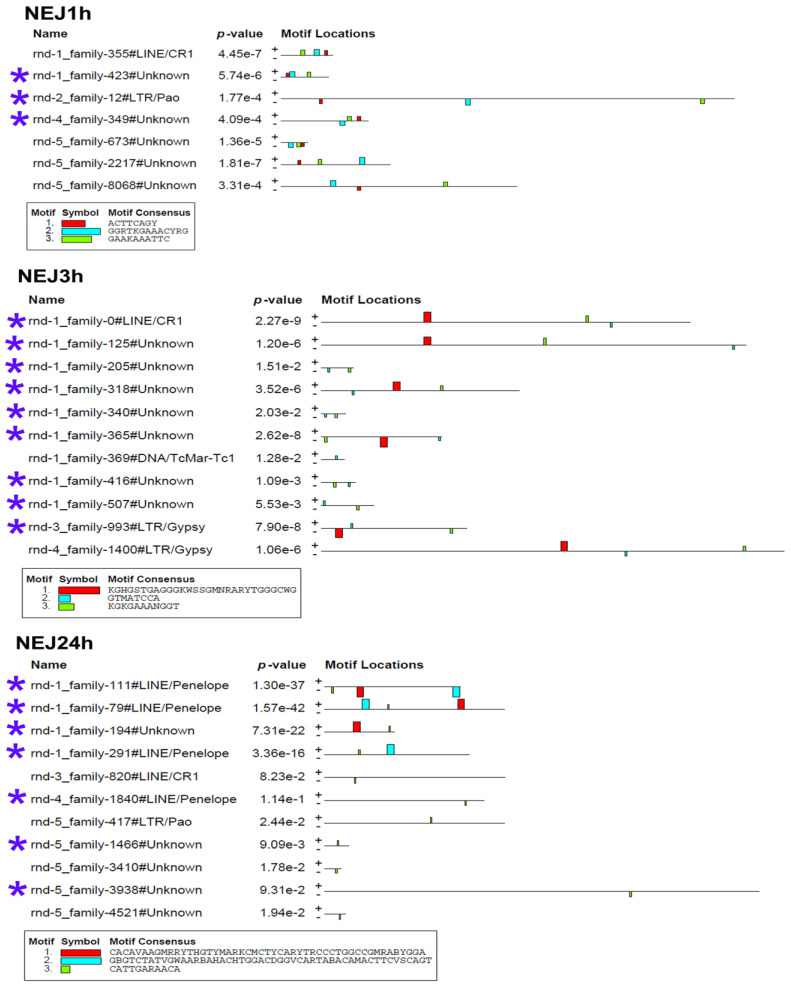
Transcription factor binding site predictions in stage-specific upregulated TE transcripts in newly emerged juveniles of 1, 3, and 24 h age. The figure is the layout showing the localization of the identified TF binding sites (colored rectangles) on the forward and reverse TE chain (labelled to the left of the schemes) with the combined match *p*-value. The block height corresponds to the site significance, i.e., taller blocks are more significant. The consensus sequences of the TF binding sites are indicated in boxes under the schemes. Blue asterisks indicate the similar lncRNA TEs.

To determine whether the stage-specific upregulated transcripts of TEs (Table S3) can presumably act as cis-regulatory elements, we had to identify the binding sites of transcription factors (TFs). We analyzed the nucleotide sequences of selected TEs using the MEME program [32], implemented in Galaxy (https://usegalaxy.eu/, last accessed 10 June 24). The results are presented in Figure 7 and Figure 8.

The MEME Suite sequential analysis of TF binding sites [32] revealed potential TF binding site (TFBS) sequences in a significant portion of upregulated TEs. Subsequent searches for known TF motifs with TomTom tool [33] confirmed the presence of several TFBS. For instance, TEs upregulated in eggs were enriched with HLH-2, Rsc30, and REF-1 sites—the transcription factors that are active during early larval development and promote hypodermal cell fusion patterning.

Additionally, juvenile TEs were found to contain sequences similar to those recognized by Zfp410 zinc-finger proteins and multifunctional Sox TF family members. In newly excysted juveniles, upregulated TEs also exhibited TFBS similar to those of Rara_DBD_3 (hormone receptor domain), retinoic acid receptors RARG and RARB, FOXG1 (fork-head DNA-binding domain), Nkx-2.9, SFFV, and the Sox TF family.

The metacercaria stage exhibited the majority of the identified TF binding sites, including those for the Sox family, Ndt80 (meiosis-specific transcription factor), HLH-2, Gm5454 (predicted protein), Etv4 (E1A enhancer binding protein), Gabpa (GA repeat binding protein), and Elk3 (member of the ETS oncogene family).

## 3. Discussion

Eukaryotic genomes contain a substantial portion of repetitive sequences, primarily composed of mobile elements. Transposons can integrate into gene regulatory regions and play a role in transcription regulation. Additionally, they serve as a source for non-coding RNAs, encompassing both short (e.g., microRNAs) and long (e.g., lncRNAs) variants [16]. Previously considered as “junk DNA”, transposons are now acknowledged as significant contributors to the structure and operation of the eukaryotic genome. This assertion finds support in various instances of transposon exaptation [23,34,35,36].

The study of the role of transposons and non-coding RNAs in the genomes of parasitic flatworms, specifically trematodes, holds significant interest. Trematodes undergo a complex life cycle, with multiple developmental stages spanning across diverse animal hosts and environments. Yet the molecular mechanisms governing stage transitions remain elusive. In recent years, research on non-coding RNAs in trematodes has garnered attention [13,15,37,38,39,40]. However, studies elucidating the role of transposons in trematodes remain relatively scarce [41,42,43,44,45,46,47]. This underscores a notable gap in our understanding, considering the pivotal role transposons play in eukaryotic genomes and their potential significance in parasite life cycles.

This work summarizes the data on repeats in the *F. hepatica* genome and is a pioneering attempt to trace the transposon transcription at different stages. In different studies, the *F. hepatica* genome, one of the largest among trematodes, exhibits varying repeat compositions, ranging from ~40% in Liverpool isolates [27] to ~50% in Oregon isolates [29,48,49]. A comprehensive analysis was conducted [49] revealing an expansion of DNA transposons (3- or 4-fold) and LTR retroelements (almost 7-fold more copies per genome) in *F. hepatica* and *F. gigantica* genomes compared to closely related Fasciolopsis buski species; *Fasciola* spp. diverged ~90 million years ago. Moreover, *F. hepatica* and F. gigantica genomes contain more intronic TEs. Despite these variations, the overall proportions of TEs remain similar across trematodes [45,50,51,52,53,54], with LINE elements prevailing in the genome (occupying between 10% and 30%) and LTR retrotransposons ranging from 4% in schistosomes to 15% in *Fasciola* spp. [48]. We suggest that a database of repeats, generated in this study with the most recent genome assembly of *F. hepatica,* has higher reliaility, considering that PacBio technology is believed to yield a better assembly of repeats compared to genome sequencing by Illumina [55]. Our analysis revealed that the mean proportion of repeats in the genome is more than 60%, with slight differences among TE groups in assemblies. Unknown elements constitute a significant portion, which causes challenges for annotation by making the program unable to assign them to known groups. Annotation of Unknown elements is a labor-intensive task and merits a separate study, given the numerous nuances involved [56,57,58]. Considering the differences in sequencing methodologies and assembly techniques, slight variations in TE composition may be attributed to assembly artifacts or intraspecific polymorphism.

A primary analysis showed that transcription of both DNA transposons and retroelements takes place at all stages, revealing sets of elements that appear to be specific to certain stages and not expressed in others. However, these TEs were not included in the list of differentially expressed repeats due to failure to pass the internal filter of the sleuth R package. In these cases, experimental validation (PCR-based) seems to be the only feasible option.

In the overall evaluation of TE expression profiles, samples were not segregated according to stages, likely due to their inherent characteristics, such as potential delays in larval development or sequencing artifacts. Notably, when looking at the expression of LTRs or Unknown elements only, sample clustering is aligned with stages, while no such pattern is observed for LINEs. Nevertheless, it has been shown that in a number of *Fasciola gigantica* genes, intronic LINEs insertions presumably contributed to the modulation of gene networks associated with the regulation of membrane transport, protein synthesis, and histone modification [49].

Futhermore, the expression of LTR retrotransposons, especially endogenous retroviruses (ERVs), is crucial for cellular processes. For instance, HERVH has been implicated in maintaining stemness in human cells [59,60] and in mice. A gradient change in transposon expression, including LTRs and LINEs, occurs during embryogenesis [61]. In contrast, other species, such as zebrafish [62] or Xenopus tropicalis [63], overwhelmingly express DNA transposons at these developmental stages. TE expression during embryogenesis has also been observed in other model organisms [64,65,66,67,68].

In the trematode life cycle, embryogenesis runs at a few stages: in the egg and in the rediae during parthenogenetic reproduction. Unfortunately, we do not know exactly what part of TE transcripts in the egg was accumulated during oogenesis and what part was newly synthesized during embryo development, since the original study [27] did not specify the timespan of RNA being isolated from eggs. RNA-seq data for parthenogenetic stages of *F. hepatica* are unavailable, making it impossible to determine TE expression levels during this stage. Likewise, significant changes occur during the metacercaria stage, where many processes are activated, including high expression levels of genes related to metabolic activity, cell signaling, DNA replication, and transcription [69,70,71,72]. Moreover, metacercariae express a set of microRNAs and lncRNAs [15,31,73]. Our data indicate that the egg and metacercariae have the highest number of specific upregulated TEs, and the putative role of these transcripts is yet to be determined.

In addition to the previously discussed TEs, lncRNAs play an important role in early development [74,75,76]. LncRNAs are known to participate in various regulatory processes, including chromatin remodeling, transcription regulation, and post-transcriptional modification [77]. Despite their low sequence homology, lncRNAs contain domains that are highly similar to TEs. These domains are assumed to be functionally significant for lncRNAs by triggering the recruitment of TFs, microRNAs, or proteins associated with chromatin modifications [78,79,80]. In our study, we identified TF binding sites within upregulated TEs, including those associated with lncRNAs. However, it is noteworthy that the presence of predicted transcription factor binding sites (TFBSs) alone does not conclusively assign a regulatory function to the TE sequences. Predicted TFBSs are often degenerate and may not fully match the canonical binding motifs. Therefore, further experimental validation of actual transcription factor binding to these sequences is required. For trematodes, this area is still in its early stages of research. For instance, in *S. mansoni*, Sox transcription factors were described only recently [81]. Nevertheless, in other organisms, such as humans and mice, it has been shown that approximately 40% of TF binding sites originate from TEs, including critical TFs like STAT1, TP53, OCT4, NANOG, and CTCF [82]. Furthermore, the interaction of transcription factors (TFs) with transposable element transcripts in various regulatory networks has been demonstrated. ZFP352 (Klf family), which binds to the SINE_B1 element in mouse embryos, facilitates the transition from the two-cell stage to further development [83]. TF Foxa1 (Forkhead family) binds to hypomethylated regions of SINE/MER1 and activates transcription of the α-fetoprotein gene (Afp) in human embryonic stem cells [84]. Human HERVs interact with numerous TFs during early human embryogenesis [85], including the Sox TF family, whose potential binding sites were identified in stage-specific upregulated TEs (Figure 7 and Figure 8).

Furthermore, stage-specific expression of TEs and their association with lncRNAs may shed light on complicated regulatory interactions, contributing to the fine-tuning of gene expression during key transitions in the trematode life cycle.

## 4. Materials and Methods

### 4.1. Genomic Data and Repetitive Element Discovery

**Figure 9:**
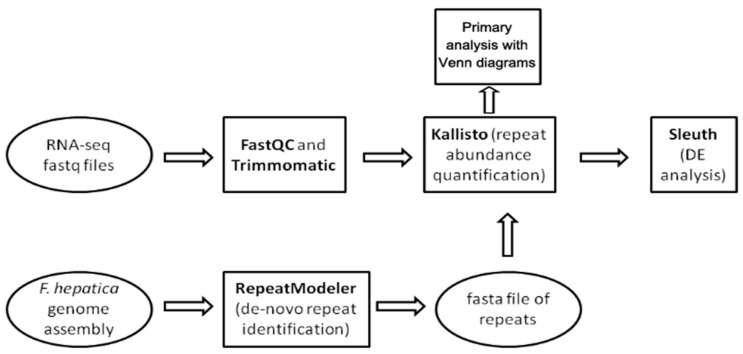
Pipeline for differential expression (DE) analysis of mobile elements within various Fasciola hepatica life cycle stages.

The workflow showing the steps of the study is provided in Figure 9. We used three public *F. hepatica* genome assemblies: GCA_900302435.1 (Bioproject PRJEB25283), GCA_002763495.2 (Bioproject PRJNA179522), and GCA_948099385.1 (Bioproject PRJEB58756). The latter was assembled in 2023 using PacBio long reads.

The de novo search for repetitive elements was performed with RepeatModeler v. 2.0.5. software [86] hosted on the Galaxy server (https://usegalaxy.eu/, last accessed 13 February 24) and GCA_948099385.1 genome assembly. RepeatModeller2 uses clustering algorithms to group similar sequences together and to identify repetitive elements de novo. Then, these clusters are used to perform multiple sequence alignments and refine the identification of repeats. For each cluster, a consensus sequence is generated, representing the common features of the sequences within the cluster, thus providing a representative model of the repetitive element. Next, repetitive elements are classified into known repeat families and the final output of a refined repeat library is generated [86]. The generated library is utilized for RepeatMasker (https://www.repeatmasker.org/, last accessed 13 April 2024) to compare the repeat content of three *F. hepatica* genome assemblies. The results are summarized in Supplementary File S1 (Table S5). In addition, we used a generated library of repeats to investigate repeat content in the transcriptome assembly (https://ftp.ebi.ac.uk/pub/databases/wormbase/parasite/releases/WBPS18/species/fasciola_hepatica/PRJEB25283/fasciola_hepatica.PRJEB25283.WBPS18.mRNA_transcripts.fa.gz, last accessed 28 February 2024) and protein coding transcripts (https://ftp.ebi.ac.uk/pub/databases/wormbase/parasite/releases/WBPS19/species/fasciola_hepatica/PRJEB58756/fasciola_hepatica.PRJEB58756.WBPS19.CDS_transcripts.fa.gz last accessed 10 June 2024) available at Wormbase Parasite. The repeat database and processing scripts are available on the GitHub page of our project (https://github.com/LisaSkalon/Mobile_elements_of_F.hepatica, last accessed 10 June 2024).

### 4.2. RNA-Seq Data Preprocessing

The study relied on publicly avaliable RNA-seq datasets for several life cycle stages of *F. hepatica* [27], with a minimum of 2 biological replicates for each stage. Raw fastq read files were downloaded from the NCBI Sequence Read Archive (Accession numbers ERR576952-ERR576969, ERR577157-ERR577160). The samples encompassed distinct developmental stages of the liver fluke, adult parasites (ad, n = 3), eggs (n = 2), metacercariae (met, n = 3), NEJ, i.e., young larvae excysted from metacercariae (1 h post-excystment: nej1h, n = 2; 3 h post-excystment: nej3h, n = 2; 24 h post-excystment: nej24h, n = 2), and 21-day liver-stage juveniles (juv; n = 2). NEJs, offspring of metacercariae, develop into juveniles and eventually mature into adults that produce eggs. A sequencing depth for each sample varies from 7058 Mb (egg1) to 15,924 Mb (NEJ24h2). A detailed list of the downloaded samples and their specifications is provided in Table S4.

The downloaded RNA-seq reads (Table S4) underwent quality control analysis using FastQC v0.12.1 [87] (Figure 9). Runs from identical samples sequenced under the same technical conditions and sharing identical BioSample names were merged using the cat Unix utility. Subsequently, reads were filtered using Trimmomatic v0.39 [88] to remove residual Illumina TruSeq adapters and eliminate low-quality bases (Phred quality score < 25).

### 4.3. Differential Expression Analysis of Transposable Elements

The expression of repetitive elements was assessed using the Kallisto v. 0.48.0 pseudoalignment-based quantification tool [89]. The output file from RepeatModeler2, containing consensus repeat sequences, served as the reference and was indexed by Kallisto. The repeat abundance for each developmental stage was quantified as transcripts per million (TPM) by aligning the set of targeted k-mers to the k-mers of the clean RNA-seq reads (with 1000 bootstrap samples).

Differential expression analysis was conducted on the Kallisto output using the sleuth R package v. 0.29.0 [90] (Figure 9). Based on the obtained data, a sample heatmap clustered by Jensen–Shannon divergence and a PCA plot were generated (Figure 3). Using the likelihood ratio test (LRT), 676 out of 1824 transposable elements with a *p*-value < 0.05 were identified as differentially expressed (Table S2, tab sleuth_significant list) following multiple comparison across all stages. Additionally, a Wald test was performed to detect false positive results for pairwise stage comparisons and reveal the fold change of TE expression.

To identify TEs expressed at specific life cycle stages, we compared lists of all the expressed TEs (with a TPM > 1 cutoff) by feeding Kallisto raw counts into Open Office Calc v. 4.1.15. Subsequently, the results were visualized using the web-based tool Venn (https://bioinformatics.psb.ugent.be/webtools/Venn/, last accessed 28 February 2024). Additionally, this tool was utilized to analyze data obtained from pairwise comparisons of stages, where the Wald test was applied. The results are presented as volcanoplots using Graphpad prism (https://www.graphpad.com/, accessed 10 June 2024) in Figure 5. We compared lists of transposons exhibiting statistically significant differences in expression, as well as separately analyzed upregulated TEs. Raw results of all described comparisons are available in Table S3. To identify TE-associated lncRNAs, a local BLAST search using Galaxy platform (https://usegalaxy.eu/last accessed 10 April 2024) was performed against the *F. hepatica* repeat database with a 70% sequence similarity parameter. The results are shown in Table S2.

### 4.4. Identification of Transcription Factor Binding Sites in Stage-Specific Upregulated TEs

Transcription factor binding sites (TFBSs) were identified in stage-specific TEs using MEME version 5.4.1 [32], implemented in the Galaxy server (https://usegalaxy.eu/, last accessed 15 June 2024). The motif search was conducted on both the forward and reverse strands, with motif widths ranging from 8 to 50 base pairs. Simple Dirichlet priors were constructed from the primary sequences, and the Zero or One Occurrence Per Sequence (ZOOPS) model was employed to identify motifs. The identified motifs were searched against the UniPROBE PBM database [91] using the Tomtom tool with E-value < 10^−3^ threshold (available at https://meme-suite.org/meme/tools/tomtom, last accessed 15 June 2024) to identify probable associated TFs.

## 5. Conclusions

Our findings regarding the expression of TEs at different life cycle stages of the *F. hepatica* trematode suggest that these transcripts play a significant role in molecular regulation. The outlined stage-specific TE expression profiles shed light on the potential TE involvement in the mechanisms regulating parasite development and adaptation to various hosts and environmental conditions. The research provides extra evidence in favor of the correlation between transposons and lncRNAs, opening new avenues to explore gene expression regulation in parasites.

## Figures and Tables

**Table 1:** List of specific TEs expressed throughout 5 stages of the *F. hepatica* life cycle.

Stage of Life Cycle	DNA-Transposons	Retroelements	Unknown Elements
Adult	-	809#LINE/CR1,1652#LINE/CR1, 2673#LINE/CR1	1132#Unk, 5212#Unk, 210#Unk, 4115#Unk, 3145#Unk, 2534#Unk, 2941#Unk, 1834#Unk, 504#Unk, 713#Unk, 392#Unk, 569#Unk, 416#Unk, 3383#Unk, 1574#Unk
Egg	-	622#LINE/CR1,596#LINE/CR1, 1595#LTR/Gypsy,456#LINE/CR1	2633#Unk, 1040#Unk,587#Unk,515#Unk, 971#Unk, 1393#Unk, 431#Unk, 1119#Unk
Juvenile	-	962#LINE/CR1, 441#LTR/Pao, 432#LINE/Penelope, 129#LINE/CR1, 313#LTR/Pao	3067#Unk, 423#Unk,764#Unk,1693#Unk, 566#Unk,9365#Unk,3346#Unk, 2338#Unk, 280#Unk,955#Unk,4831#Unk,138#Unk,584#Unk, 196#Unk, 335#Unk,297#Unk, 246#Unk
Metacercaria	487#DNA/CMC-EnSpm, 2355#DNA/TcMar-Tigger	6300#LTR/Gypsy, 191#LINE/CR1	969#Unk,1704#Unk,1134#Unk,1234#Unk, 371#Unk, 867#Unk, 1263#Unk,1123#Unk, 38#Unk,1339#Unk,495#Unk,426#Unk,69#Unk
NEJ 24 h	-	467#LTR/Gypsy, 183#LINE/CR1, 2677#LINE/CR1, 502#LINE/CR1	410#Unk, 336#Unk, 2170#Unk, 3876#Unk, 6772#Unk, 1365#Unk

Names of repeats were shortened, number before TE name indicates the family ID, detected by RepeatModeler2, and raw results are in Table S1; Unk—Unknown.

**Table 2:** List of TEs selectively expressed in 7 stages of the *F. hepatica* life cycle.

Stage of Life Cycle	DNA-Transposons	Retroelements	Unknown Elements
Adult	-	809#LINE/CR1	1132#Unk, 5212#Unk, 210#Unk, 504#Unk, 2534#Unk, 2941#Unk, 713#Unk, 392#Unk, 569#Unk, 416#Unk, 3383#Unk, 1574#Unk,1834#Unk,
Egg	-	622#LINE/CR1,596#LINE/CR1, 1595#LTR/Gypsy	2633#Unk, 1040#Unk, 587#Unk, 515#Unk, 971#Unk, 1393#Unk, 431#Unk, 1119#Unk
Juvenile	-	313#LTR/Pao, 962#LINE/CR1 441#LTR/Pao, 129#LINE/CR1	3067#Unk, 764#Unk, 1693#Unk 566#Unk,9365#Unk,3346#Unk, 2338#Unk, 280#Unk,955#Unk 4831#Unk, 584#Unk, 196#Unk 335#Unk, 297#Unk, 246#Unk
Metacercaria	2355#DNA/TcMar-Tigger	-	969#Unk, 704#Unk, 134#Unk, 234#Unk, 371#Unk, 867#Unk, 1339#Unk, 495#Unk, 426#Unk, 69#Unk
Juvenile 24 h	-	467#LTR/Gypsy, 183#LINE/CR1 2677#LINE/CR1, 502#LINE/CR1	410#Unk, 336#Unk, 3876#Unk 1365#Unk
Juvenile 1 h	-	1677#LTR/Gypsy	261#Unk, 4259#Unk
Juvenile 3 h	-	-	1270#Unk, 892#Unk, 547#Unk

Names of repeats were shortened, number before TE name indicates the family ID, detected by RepeatModeler, and raw results are in Table S1; Unk—Unknown.

## Data Availability

All the data are presented in Supplementary Files and hosted on Github (https://github.com/LisaSkalon/Mobile_elements_of_F.hepatica, last accessed 10 June 2024).

## Supplementary Materials

The following supporting information can be downloaded at: https://www.mdpi.com/article/10.3390/ncrna10040039/s1, Supplementary File S1: Table S5: *F. hepatica* genome content comparison, and Table S6: Juveline TE expression; Table S1: Raw results of expressed repeat comparisons, Table S2: Differential expression analysis and lncRNA BLAST search; Table S3: Wald test results; Table S4: list of samples.
