# Supplementary material for: Expression of Transposable Elements throughout the Fasciola hepatica Trematode Life Cycle
Source: Noncoding RNA. 2024 Jul 3;10(4):39. doi: 10.3390/ncrna10040039 (PMC11270206; doi:10.3390/ncrna10040039)
Supplement: Supplementary file 1 [file ncrna-10-00039-s001.zip › Supplementary File S1_updated.pdf]

Supplementary File S1. F.hepatica genome assemblies' content comparison and juveline TE expression.

Table S5. Comparison of repeat content in three *F. hepatica* genomes —Oregon isolate (PRJNA179522) and 2 Liverpool isolates (PRJEB25283 and PRJEB58756– latest assembly).

	F.hepatica Oregon isolate (PRJNA179522)		*F.hepatica* Liverpool isolate (PRJEB25283)		*F.hepatica* Liverpool isolate (PRJEB58756)		**Mean %**
	**Number of elements***	**% of sequence**	**Number of elements***	**% of sequence**	**Number of elements***	**% of sequence**	
**Retro-elements**	766200	40.69	814955	41.87	1106573	41.53	**41.36**
**SINEs**	27136	0.41	27906	0.39	30486	0.35	**0.38**
**LINEs**:**	552927	29.64	583497	30.26	837331	29.12	**29.67**
**L2/CR1/Rex**	260093	15.28	276438	15.83	441440	15.84	**15.65**
**R1/LOA/Jockey**	387	0.014	407	0.023	434	0.01	**0.015**
**R2/R4/NeSL**	2767	0.1	2898	0.1	3333	0.1	**0.1**
**RTE/Bov-B**	157438	9.66	163005	9.74	169597	8.67	**9.35**
**L1/CIN4**	1164	0.02	1241	0.02	1421	0.03	**0.023**
**LTR elements:**	186137	10.64	203552	11.22	238756	12.06	**11.3**
**BEL/Pao**	64013	4.84	73432	5.29	90421	6.27	**5.46**
**Gypsy/DIRS1**	122056	5.79	130043	5.93	148228	5.79	**5.83**
**Retroviral**	0	0	77	0.004	107	0.002	**0.003**
**DNA transposons**	66406	2.51	69852	2.48	77170	2.22	**2.4**
**hobo-Activator**	3074	0.21	3214	0.21	3663	0.19	**0.2**
**Tc1-IS630-Pogo**	40943	1.12	42756	1.08	47664	0.97	**1,05**
**MULE-MuDR**	2152	0.11	2496	0.12	2878	0.12	**0.116**
**PiggyBac**	2556	0.09	2631	0.08	2969	0.07	**0.08**
**Rolling-circles**	6228	0.18	6707	0.18	7965	0.18	**0.18**
**Unclassified**	957753	19.81	1015696	19.84	1582003	22.57	**20.74**
**Total interspersed repeats**	63.01		64.18		66.32		**64.5**
**Small RNA**	13832	0.23	14312	0.22	17291	0.66	**0.37**
**Satellites**	128	0.002	131	0.002	1711	0.03	**0.011**
**Simple repeats**	91500	0.42	121234	0.6	175690	1.95	**0.99**
**Low complexity**	2572	0.02	2671	0.02	3880	0.02	**0.02**

*most repeats fragmented by insertions or deletions have been counted as one element

**LINE elements include Penelope transposons

Table S6. List of TEs selectively expressed in juvenile stages of *F.hepatica* lifecycle.

Stage of life cycle	DNA-transpo-sons	Retroelements	Pseudo-genes	Unknown
**Juve-nile (21 day)**	1876#DNA/CMC-EnSpm, 3201#DNA/TcMar-Pogo<br>380#DNA	962#LINE/CR1, 448#LINE/Penelope, 2#LINE/CR1, 557#LINE/CR1,1591#LTR/Gypsy, 125#LINE/CR1, 441#LTR/Pao, 467#LINE/CR1, 963#LINE/CR1, 77#LTR/Gypsy 129#LINE/CR1, 518#LTR/Pao, 1248#LTR/Pao, 2372#LTR/Pao, 484#LINE/Rex-Babar, 1622#LTR/Gypsy,459#LINE/CR1, 771#LINE/CR1, 2922#LTR/Pao, 313#LTR/Pao, 704#LTR/Pao,1036#LINE/Pene-lope,117#LINE/CR1,114#LINE/RTE-RTE, 547#LTR/Gypsy	-	3067#Unk, 544#Unk,272#Unk, 1971#Unk,592#Unk,4222#Unk371#Unk, 620#Unk, 112#Unk, 591#Unk, 3288#Unk,764#Unk, 1395#Unk, 866#Unk,548#Unk, 1229#Unk,9364#Unk, 3282#Unk, 1693#Unk 566#Unk, 556#Unk, 590#Unk, 9365#Unk, 3142#Unk, 1134#Unk, 863#Unk, 3346#Unk, 2338#Unk, 4611#Unk, 280#Unk, 4162#Unk, 955#Unk, 4831#Unk, 572#Unk, 230#Unk, 584#Unk, 196#Unk, 588#Unk, 41#Unk, 356#Unk, 335#Unk, 720#Unk, 508#Unk, 297#Unk, 246#Unk, 2210#Unk, 5305#Unk
**NEJ 1h**	-	191#LINE/CR1, 1677#LTR/Gypsy, 617#LINE/CR1, 1930#LINE/CR1	-	614#Unk, 261#Unk, 4115#Unk, 3145#Unk, 1263#Unk, 585#Unk, 4259#Unk
**NEJ 3h**	-	2673#LINE/CR1	4008#tRNA	4019#Unk, 1270#Unk, 892#Unk, 1123#Unk, 547#Unk, 3850#Unk
**NEJ 24h**	-	467#LTR/Gypsy, 183#LINE/CR1, 5510#LINE/CR1-Zenon, 2677#LINE/CR1, 502#LINE/CR1	-	410#Unk, 1185#Unk, 932#Unk, 336#Unk, 1680#Unk, 284#Unk, 3876#Unk, 1365#Unk, 1658#Unk

- Unk — Unknown transposons, NEJ – newly excysted juvenile,
